# Equity of travel to access surgery and radiation therapy for lung cancer in New Zealand

**DOI:** 10.1007/s00520-024-08375-9

**Published:** 2024-02-20

**Authors:** Jason Gurney, Anna Davies, James Stanley, Jesse Whitehead, Shaun Costello, Paul Dawkins, Kimiora Henare, Christopher G. C. A. Jackson, Ross Lawrenson, Nina Scott, Jonathan Koea

**Affiliations:** 1 University of Otago Wellington, Newtown, PO Box 7343, Wellington, 6242 New Zealand; 2 University of Waikato, Hamilton, New Zealand; 3 Te Whatu Ora – Southern, Dunedin, New Zealand; 4 Te Whatu Ora – Counties Manukau, Auckland, New Zealand; 5 University of Auckland, Auckland, New Zealand; 6 Department of Medicine, University of Otago, Dunedin, New Zealand; 7 Population and Public Health, Te Whatu Ora – Waikato, Hamilton, New Zealand; 8 Te Aka Whai Ora, Hamilton, New Zealand; 9 Te Whatu Ora – Waitematā, Auckland, New Zealand

**Keywords:** Lung cancer, Treatment, Surgery, Radiation therapy, Indigenous, Māori, Travel, Disparities, Equity

## Abstract

**Purpose:**

Centralisation of lung cancer treatment can improve outcomes, but may result in differential access to care for those who do not reside within treatment centres.

**Methods:**

We used national-level cancer registration and health care access data and used Geographic Information Systems (GIS) methods to determine the distance and time to access first relevant surgery and first radiation therapy among all New Zealanders diagnosed with lung cancer (2007–2019; *N* = 27,869), and compared these outcomes between ethnic groups. We also explored the likelihood of being treated at a high-, medium-, or low-volume hospital. Analysis involved both descriptive and adjusted logistic regression modelling.

**Results:**

We found that Māori tend to need to travel further (with longer travel times) to access both surgery (median travel distance: Māori 57 km, European 34 km) and radiation therapy (Māori 75 km, European 35 km) than Europeans. Māori have greater odds of living more than 200 km away from both surgery (adjusted odds ratio [aOR] 1.83, 95% CI 1.49–2.25) and radiation therapy (aOR 1.41, 95% CI 1.25–1.60).

**Conclusions:**

Centralisation of care may often improve treatment outcomes, but it also makes accessing treatment even more difficult for populations who are more likely to live rurally and in deprivation, such as Māori.

**Supplementary Information:**

The online version contains supplementary material available at 10.1007/s00520-024-08375-9.

## Introduction

New Zealand is similar in area to the UK, but with a much smaller population—around 5 million in New Zealand, compared to 69 million in the UK [1]. Nearly 20% of the New Zealand population lives rurally [2]. New Zealand’s small and geographically dispersed population makes providing healthcare close to where people live an ongoing national challenge [3].

Nearly all lung cancer surgery and radiation therapy in New Zealand is delivered in only 6 regional cancer centres (Auckland, Hamilton, Palmerston North, Wellington, Christchurch, Dunedin), and not all of these will provide all aspects of required care [4]. This small number of cancer treatment centres, combined with the geographic dispersion of the population, means that New Zealanders need to travel (often far) to access treatment for their lung cancer.

New Zealand’s Indigenous Māori population are disproportionately impacted by this situation. Māori are more likely to live in rural areas (25%, compared to 20% of the majority European population, 7% Pacific, and 5% Asian), and 32% of those who live in the most remote areas of New Zealand are Māori [2], despite Māori comprising around 17% of the general population [5]. This has consequences for treatment access: in a recent liver cancer study, we found that Māori, on average, had to travel 120 km (over 2 h) to access their first liver cancer surgery, compared to around 60 km (less than 1 h) for those of European ethnicity [6]. Māori are also more likely to be diagnosed with poor-prognosis cancers (like liver cancer) that require complex treatment [7], with this treatment only delivered in a few locations [6]. Māori are also much more likely to live in areas of high deprivation than Europeans [8]—meaning that New Zealand’s Indigenous population face the dual issue of being more likely to travel long distances to access treatment, but less likely to be able to afford that travel.

Lung cancer is among the most commonly-diagnosed and the most common cause of cancer death for Māori in New Zealand [7], and a major driver of the 8-year gap life expectancy between Māori and non-Māori New Zealanders. Like liver cancer, treatment complexity for lung cancer means that surgery and radiation therapy mostly occurs in those large cancer centres with the facilities and clinicians required to deliver the required care. Also like liver cancer, this suggests Māori are likely to need to travel further to access care than Europeans; however, this travel disparity remains unexplored for lung cancer.

There is evidence from several other cancers that outcomes can be best optimised when treatment is delivered in high-volume facilities in metropolitan centres [9, 10]. The increased likelihood for Māori to live outside these centres, alongside reduced capacity to afford direct and indirect costs of travelling to main centres, could be a partial driver of disparities in lung cancer survival between Māori and other ethnic groups in New Zealand [11]. Understanding the extent to which Māori access lung cancer treatment in high-volume centres relative to Europeans could reveal a focal point for quality improvement.

The study aims to examine inequities in travel to access lung cancer surgery and radiation therapy for Māori in New Zealand compared to the majority European population, including distance travelled to access first surgery and radiation therapy, and the estimated travel time. We also examine inequities in treatment within high-volume centres for two of the most common curative surgical interventions for lung cancer.

## Methods

### Participants and data sources

This study included all lung cancer registrations (ICD-10-AM code: C33-C34) diagnosed in New Zealand from 2007 to 2019, derived from the New Zealand Cancer Registry (NZCR; *N* = 27,869, 5,601 Māori, 1,267 Pacific, 1,180 Asian, 123 MELAA/Other, 19,698 European). The NZCR is a national record of all malignancies diagnosed in New Zealand, with the exception of basal and squamous cell cancers of the skin [12]. NZCR data were linked nationally to all public and reporting private hospital inpatient records (National Minimum Dataset, NMDS), as well as emergency department and outpatient records (National Non-Admitted Patients Collection, NNPAC), Primary Health Organisation enrolment data (PHO Enrolment Collection), and the Mortality Collection.

### Demographic variables

Age at diagnosis was derived from the NZCR, and categorised as < 50, 50–64, 65–74, and 75 + . Sex was also derived from the NZCR (all records were either male or female). Prioritised ethnicity was derived from the NZCR and other National Collections datasets, and categorised as either Māori, Pacific, Asian, European, or MELAA/Other. This study focuses on Māori and European populations: We have presented data on treatment receipt for all ethnic groups in the Supplementary Material, but have not presented data on travel distance/time or hospital volume for ethnic groups other than Māori or European due to data sparseness.

Comorbidity was defined at time of lung cancer diagnosis using the C3 cancer comorbidity index. This index uses inpatient hospitalisation data (NMDS) for the 5-year period prior to diagnosis, looks for ICD codes for diagnoses of 42 individual conditions, and then derives a weighted score based on the relationship of each condition with non-cancer mortality in a cancer population [13]. For descriptive analysis, C3 scores were categorised as ‘0’ (score <  = 0), ‘1’ (> 0, <  = 1), ‘2’ (> 1, <  = 2) and ‘3’ (> 2). Those with no recorded comorbidity were assigned a score of 0 [13]. The C3 index was used as a categorical variable for descriptive analysis, and modelled in logistic regression using restricted cubic splines with three knots [14].

### Treatment variables

Receipt of primary surgery was defined using publicly funded inpatient hospitalisation data. Procedures were extracted using Australasian College of Health Informatics (ACHI) ICD-10-AM codes (2nd Edition), with relevant curative and palliative procedures identified by our clinical advisors (Supplementary Material 1). We included all surgical procedures occurring up to 90 days prior to date of diagnosis and up to one year after lung cancer diagnosis. We included this 90-day pre-diagnosis period to allow for delays in diagnosis and/or diagnosis following a relevant procedure. Receipt of radiation therapy was defined using the publicly funded inpatient and outpatient hospitalisation data (Supplementary Material 2). We identified first receipt of radiation therapy for each individual in the period 90 days before diagnosis and up to one year after lung cancer diagnosis.

### Travel variables

To compare distance travelled to access first primary surgery and/or radiation therapy, we used Geographic Information Systems (GIS) analysis to determine the distance in kilometres between the location where a patient lived at the time of their procedure, and the location where their procedure occurred. To do this, we derived the domicile code of patient residence [15] at the time of their first surgery and/or radiation therapy and the geocoded coordinates of the facility where they underwent their procedure [16]. Other geographic datasets used in the analysis included Beer’s road network layer [17], Statistics New Zealand’s Census Area Units [18, 19], and the Land Information New Zealand street address dataset [20] (used to create address-weighted centroids for each domicile code).

The OD-Matrix function within the GIS software programme ArcGIS (Environmental Systems Research Institute, USA) was used to estimate distance and travel times to surgery and/or radiation therapy. To do this, we estimated the road network distance and travel time between the address-weighted centroid of each patient’s domicile of residence, and the geocoded coordinates of the facility where a procedure took place. This analysis used a street network layer developed by Beere [17] to accurately estimate both travel distance and time. Using this method, travel time is calculated as being the fastest route assuming free-flow traffic, taking into account road type including the availability of highways, the number of lanes, road surface type, and estimated average speed given these factors. In this way, travel time provides a more ‘real world’ estimate of travel required to access services such as health care [17].

Missing data prevented the attribution of distance or travel time for 8 registrations for first primary surgery (0.2%) and 16 registrations for radiation therapy (0.1%) across the whole cohort. One-way distance from domicile of residence to location of treatment by road was expressed in kilometres (km), and categorised as < 25 km, 25–99 km, 100–199 km, and > 200 km to represent a range of typical travelling patterns: close or across-town travel (< 25 km), travel from surrounding districts (25–99 km), regional travel from nearby cities or towns (100–199 km) and inter-regional travel (> 200 km). One-way travel time was expressed in minutes (min), and categorised as < 60 min, 60–149 min, and > 150 min. These travel times represent close, across-town or surrounding district travel (< 60 min), regional travel from nearby cities or towns (60–149 min, or 1–2.5 h), and inter-regional travel (> 150 min, or more than 2.5 h). In this way, travel time was minimised to three categories instead of four, to maximise the meaningfulness of these category definitions in the New Zealand road travel context.

### Hospital volume variable

To investigate whether there were disparities in access to high-volume surgical facilities for the two most common curative surgical procedures (lobectomy and segmental resection), we extracted data from the NMDS for every lobectomy and segmental resection nationwide (i.e. in the total population, not just the cohort for this study). We then used facility codes from the NMDS to determine the facility where each procedure took place (e.g. Wellington Regional Hospital). We then created frequency tables for each procedure, and ranked facilities on how frequently a given procedure was performed. We then categorised each facility as high-, medium-, or low-volume for each procedure, with demarcations between categories made where the data showed clear breaks in terms of volume. A full list of the facilities, procedure frequencies, the relevant volume category and a rationale for setting specific category boundaries is included in Supplementary Material 3.

### Statistical analysis

For descriptive analysis, we calculated frequencies and both crude (i.e. unadjusted) and age-standardised proportions, stratified by ethnicity. Direct age-standardised proportions were determined in SAS v9.4 (SAS Institute Inc., USA) using the *proc stdrate* procedure, using the total Māori lung cancer population from the analysis cohort as the standard population [21]. We also determined the median travel distance in kilometres and the median travel time in minutes, and calculated an interquartile range (IQR) for both travel measures.

To compare travel distance, travel time and hospital volume category between Māori and Europeans, we used logistic regression (SAS procedure *proc logistic*), with European as the reference group. We compared Māori and European on the odds of a) belonging to a certain travel distance category (e.g. < 25 km); b) belonging to a certain time category (e.g. < 60 min), or c) belonging to a certain hospital volume category (e.g. high-volume). Odds ratios (OR) and their 95% confidence intervals were extracted from crude (i.e. unadjusted) models, as well as models adjusting for age, sex, type of first primary treatment (e.g. lobectomy) (for surgical treatments only) and comorbidity (C3 index). Age and sex were included as classic confounders; surgical procedure type was included to control for possible differences in the types of procedures undertaken among Māori vs. Europeans; and comorbidity was included control for patient morbidity at the time of treatment, which could feasibly influence treatment complexity and hence the location where treatment can be undertaken.

This study was performed in line with the principles of the Declaration of Helsinki. Approval was granted by the University of Otago Human Ethics Committee (reference # HD18/056). Data management and analysis was conducted in Microsoft Excel (Microsoft Corporation, U.S.A.) and SAS v9.4 (SAS Institute, U.S.A.).

## Results

Descriptive results for receipt of surgery and radiation therapy are shown in Table 1. Fewer Māori received any primary surgery for lung cancer (age-standardised percentage: Māori 13%, European 19%), with this difference most evident for curative surgery (Māori 9%, European 14%). Māori and European were similarly likely to receive radiation therapy once adjusted for age (Māori and European 44%).

**Table 1:** Receipt of surgery and radiation therapy, for Māori and Europeans with lung cancer

	Māori			European		
Treatment	*n*	*%*	*Age Std. %*	*n*	*%*	*Age Std. %*
Received Any Primary Surgery	745	13%	13%	3073	16%	19%
Received Any Curative Surgery	522	9%	9%	2264	11%	14%
Lobectomy	345	6%	6%	1510	8%	9%
Segmental Resection	163	3%	3%	725	4%	4%
Pneumonectomy	37	1%	1%	161	1%	1%
Other Curative	4	0%	0%	18	0%	0%
Received Any Palliative Surgery	241	4%	4%	881	4%	5%
Drainage	76	1%	1%	177	1%	1%
Pleurodesis	157	3%	3%	685	3%	4%
Other Palliative	18	0%	0%	49	0%	0%
Received Any Radiation Therapy	2446	44%	44%	7620	39%	44%

*Age Std.* = Age standardised to the Māori cohort

Travel distance and travel time to first surgery and first radiation therapy is shown in Table 2 for Māori compared to European. Median travel distance for Māori was 23 km (or 68%) further to receive their first primary surgery (median distance: Māori 57 km, IQR 15–193 km; Europeans 34 km, IQR 11–142 km). Māori were less likely to live less than 2 km from their surgery (age-standardised percentage: Māori 37%, European 43%; adjusted odds ratio [OR] 0.76, 95% CI 0.64–0.90), and commensurately more likely to live more than 200 km from their surgery (Māori 24%, European 16%; adj. OR 1.83, 95% CI 1.49–2.25). This tendency for Māori to need to travel further for their surgery was reflected in travel time, with Māori needing to travel for 19 min (or 48%) longer than European (median time: Māori 59 min, IQR 22–161 min; European 40 min, IQR 18–123 min). Consequently, Māori were less likely to travel for less than 60 min (Māori 50%, European 58%; adj. OR 0.69, 95% CI 0.58–0.81) and more likely to travel 150 min or more (Māori 27%, European 19%; adj. OR 1.63, 95% CI 1.34–1.98).

**Table 2:** Distance and travel time to first surgery and first radiation therapy for Māori compared to Europeans

	Māori			European			Odds Ratios (95% CI)	
Travel to Treatment	*n*	*%*	*Age Std. %*	*n*	*%*	*Age Std. %*	*Crude*	*Adjusted*
Distance to First Primary Surgery (Km):								
< 25 km	282	38%	37%	1354	44%	43%	0.77 (0.66–0.91)	0.76 (0.64–0.90)
25–100 km	144	19%	20%	688	22%	23%	0.83 (0.68–1.02)	0.83 (0.68–1.02)
100–200 km	140	19%	19%	564	18%	18%	1.03 (0.84–1.26)	1.05 (0.85–1.30)
200 + km	178	24%	24%	463	15%	16%	1.77 (1.46–2.15)	1.83 (1.49–2.25)
Missing location information	1	0%	0%	4	0%	0%	-	-
Median in Kilometres (IQR)	57 km (15—193)			34 km (11—142)				
Travel Time to First Primary Surgery (Min)								
< 60 min	375	50%	50%	1799	59%	58%	0.72 (0.61–0.84)	0.69 (0.58–0.81)
60–150 min	169	23%	23%	698	23%	23%	1 (0.82–1.21)	1.04 (0.86–1.27)
150 + min	200	27%	27%	572	19%	19%	1.6 (1.33–1.93)	1.63 (1.34–1.98)
Missing location information	1	0%	0%	4	0%	0%	-	-
Median in Minutes (IQR)	59 min (22—161)			40 min (18—123)				
Distance to First Radiation Therapy (Km):								
< 25 km	806	33%	33%	3304	43%	42%	0.64 (0.58–0.71)	0.66 (0.60–0.73)
25–99 km	575	24%	23%	1849	24%	24%	0.96 (0.86–1.07)	0.95 (0.85–1.06)
100–199 km	590	24%	24%	1354	18%	18%	1.47 (1.32–1.64)	1.41 (1.26–1.59)
200 + km	472	19%	20%	1102	14%	15%	1.41 (1.26–1.59)	1.41 (1.25–1.60)
Missing location information	3	0%	0%	11	0%	0%	-	-
Median in Kilometres (IQR)	75 km (17–167)			35 km (10–149)				
Travel Time to First Radiation Therapy (Min):								
< 60 min	1130	46%	46%	4491	59%	58%	0.6 (0.55–0.66)	0.62 (0.56–0.68)
60–149 min	778	32%	32%	1819	24%	24%	1.49 (1.35–1.64)	1.45 (1.30–1.61)
150 + minutes	535	22%	22%	1299	17%	17%	1.36 (1.22–1.53)	1.34 (1.19–1.51)
Missing location information	3	0%	0%	11	0%	0%	-	-
Median in Minutes (IQR)	69 min (26–138)			41 min (17–125)				

*Age Std.* = Age standardised to the Māori cohort. Adjusted ORs for surgery adjusted for age (categorical variable), sex, comorbidity (splined variable) and surgery category. Adjusted ORs for radiation therapy adjusted for age (categorical variable), sex and comorbidity (splined variable)

Similarly, for first radiation treatment Māori needed to travel 40 km (or 114%) further as median distance (median distance: Māori 75 km, IQR 17–167 km; European 35 km, IQR 10–149 km). Māori were less likely to live less than 25 km from their radiation therapy location (age-standardised percentage: Māori 33%, European 42%; adj. OR 0.66, 95% CI 0.60–0.73), and commensurately more likely to live more than 200 km from their surgery (Māori 20%, European 15%; adj. OR 1.41, 95% CI 1.25–1.60). These differences were reflected in travel time, with Māori needing to travel for 28 min (or 68%) longer than Europeans (median time: Māori 69 min, IQR 26–138 min; Europeans 41 min, IQR 17–125 min). Consequently, Māori were less likely to travel for less than 60 min (Māori 46%, European 58%; adj. OR 0.62, 95% CI 0.56–0.68) and more likely to travel for 150 min or more (Māori 22%, European 17%; adj. OR 1.34, 95% CI 1.19–1.51).

A comparison of the surgical volume of hospitals where Māori underwent a lobectomy or segmental resection compared to European is shown in Table 3. Māori were more likely to undergo a lobectomy at a high-volume facility compared to European (age-standardised percentage: Māori 53%, European 41%; adj. OR 1.67, 95% CI 1.31–2.13) and commensurately less likely to undergo the procedure in a medium-volume facility (Māori 45%, European 55%; adj. OR 0.63, 95% CI 0.49–0.80). Treatment at a low-volume facility was similar between groups (Māori 2%, European 3%; ORs not calculated due to sparse data). Māori were also more likely to undergo a segmental resection at a high-volume facility compared to Europeans (Māori 31%, European 20%; adj. OR 1.68, 95% CI 1.10–2.57), and marginally less likely to undergo the procedure in a medium-volume facility (Māori 64%, European 75%; adj. OR 0.63, 0.42–0.94). Treatment at a low-volume facility was similar between groups (Māori 5%, European 5%; ORs not calculated due to sparse data).

**Table 3:** Comparison of surgical volume of hospitals where patients underwent a lobectomy or segmental resection, for Māori compared to European

	Māori			European			Odds Ratios (95% CI)	
Hospital Volume	*n*	*%*	*Age Std. %*	*n*	*%*	*Age Std. %*	*Crude*	*Adjusted*
*Lobectomy*								
High Volume	181	53%	53%	629	43%	41%	1.55 (1.22–1.96)	1.67 (1.31–2.13)
Medium Volume	151	45%	45%	804	54%	55%	0.68 (0.53–0.86)	0.63 (0.49–0.80)
Low Volume	7	2%	2%	47	3%	3%	-	-
*Segmental Resection*								
High Volume	44	31%	31%	121	20%	20%	1.81 (1.20–2.72)	1.68 (1.10–2.57)
Medium Volume	90	64%	64%	453	75%	75%	0.59 (0.40–0.87)	0.63 (0.42–0.94)
Low Volume	7	5%	5%	30	5%	5%	-	-

*Age Std.* = Age standardised to the Māori cohort. Adjusted ORs adjusted for age (categorical variable), sex and comorbidity (splined variable)

## Discussion

In this study, we found that Māori with lung cancer tend to need to travel further (with longer travel times) to access both surgery and radiation therapy than Europeans with lung cancer. We also found that Māori are more likely to undergo lung cancer surgery (lobectomy and segmental resection) within a high-volume facility than Europeans.

As noted in the Introduction, evidence suggests outcomes from cancer treatment may be best when patients are treated in high-volume, centralised facilities [9, 10]. Concentrating the greatest expertise into a few centres, as opposed to spreading this expertise across more locations, should logically improve care and outcomes for those who are able to access timely treatment in those few centres [22]. Specific to lung cancer, being admitted to a specialist lung cancer treatment hub increases the likelihood of curative surgery, and consequently improves survival outcomes [23]. But as with many elements of cancer service planning, there is a tension between the need to centralise care to maximise positive outcomes against the need to provide care close to home. Alongside this tension is the growing evidence that centralisation of services makes accessing care more difficult for some populations more than others—as clearly shown for Māori in the current study, and in other similar international studies noted above.

Entwined with our observation that Māori tend to need to travel further to access lung cancer treatment, is our finding that Māori were much more likely to have a lobectomy or segmental resection surgery in a high-volume facility (Table 3). By way of background, one hospital in New Zealand’s largest city (Auckland) was the sole high-volume facility for both lobectomies and segmental resections. Māori are more likely to need to travel from across a wide catchment to access care at this high-volume facility, from the top of North Island of New Zealand to Auckland’s southern districts, and beyond. The tendency for Māori to be treated at this high-volume facility is positive in terms of likely outcomes from treatment, for the reasons outlined above. Simultaneously, it is likely that this increased propensity to be treated at a high-volume facility is also contributing to the observed disparity in the travel required to access surgery relative to Europeans. In order to avoid creating or exacerbating inequities in access to care, there is a need to ensure that marginalised populations who are more likely to live rurally (such as Māori in New Zealand) are well-supported to overcome barriers to accessing timely centralised care. Such support might be delivered by up-front funding (rather than relying on reimbursement systems) to assist with travel-related costs, including providing funding for a support person to accompany the patient [24]. Relatedly, there is also a need for more culturally appropriate health support workers (such as Māori cancer care navigators [4]) to help marginalised populations navigate their care [3]: this may not reduce the distance to care, but it will likely improve access to travel support, and help to make the care pathway as clearly-marked as possible. We have also noted previously [6] that there is a need to shift clinics that require attendance in the lead-up to and following surgery closer to home for the patient, rather than requiring a patient to travel across regions for a short pre-surgery appointment that could have been held via telehealth or remote clinic.

We recently observed that while Māori are less likely to access lung cancer surgery, there is no apparent difference in the timing of that surgery relative to diagnosis—suggesting that the additional travel barriers experienced by Māori are not necessarily translating into extended wait times for treatment [25]. This suggests significant resilience within Māori to overcome the barriers to care highlighted in this manuscript. However, we do not know the extent to which these barriers are preventing treatment access in the first place, because this study examined distance and travel time to lung cancer treatment among those who actually received treatment. It does not examine the extent to which distance to treatment acts as a barrier to receiving treatment. In Australia, a greater proportion of patients with early-stage lung cancer who lived 100 km or more from a specialist treatment hub did not have surgery (51%) than patients living 0–39 km away (38%) [26]. In Canada, the rate of radiation therapy receipt within 1 year of lung cancer diagnosis reduces with increasing distance from radiation therapy treatment centre—with the odds decreasing by 16% after 20 min, and then by a further 6% per hour thereafter [27]. In the US, longer travel times to access treatment for early-stage lung cancer are actually associated with a higher proportion of patients accessing best-practice treatment [28]—this resonates with our finding that Māori may be travelling further than Europeans in order to access treatment at a high-volume facility. However, the US study also found that increased travel time for Black or Filipino patients was associated with reduced access to best-practice treatment [28]. In the UK, travel distance amplifies the impact of deprivation—in other words, being poor acts as a barrier to treatment, and that barrier becomes larger the further away you live from where treatment is provided [29, 30]. This scenario could well apply to Māori, and these travel-related barriers may be at least partially driving the lower surgery rates (shown in Table 1). However, to evaluate this more fully, we would need to examine the potential travel required for all lung cancer patients who may be candidates for surgery, identify those who received surgery and those who did not, and examine whether this distance varies by ethnicity. We aim to complete this analysis in due course.

While centralisation of care can improve care quality and thus outcomes, we have shown here and elsewhere [6] that this centralisation makes accessing care disproportionately more difficult for Māori than it does for other groups. The evidence presented in this manuscript emphasises the need for new funding models to better support Māori to access centralised cancer treatment, as well as the need for new models of care that improve the availability, affordability and acceptability of cancer treatment for our Indigenous peoples. Based on our findings and other previous evidence, we recommend the following actions:

- Deliver those aspects of lung cancer treatment that can be decentralised (such as pre-surgery appointments) via telehealth or in physical locations close to where Māori live, wherever feasible;
- Where travel is required, provide financial support up-front to cover transport and accommodation costs, and provide accommodation for patients and whanau as part of standard care (currently only provided under certain circumstances in New Zealand [24]);
- Substantially expand the number of Māori cancer care navigators, to maximise the support available to Māori with lung cancer to navigate the often-complex (and geographically-distant) lung cancer treatment pathway [4].

### Strengths and limitations

We used national-level data on surgery and radiation therapy linked to national cancer registry data, maximising the generalisability of our findings to the New Zealand population. Due to the nature of these data, we were unable to discern the types of radiation therapy, or whether this therapy was delivered with curative or palliative intent. Because data on privately funded hospitalisations were incomplete (private hospitals are not required to report privately-funded treatment to the NMDS), we did not include privately funded treatment within this study.

We have inferred from our findings that the primary driver of the observed difference in travel time and distance, as well as access to high-volume hospitals, is differences between Māori and Europeans with lung cancer in terms of where they tend to live relative to where treatment is located. While this is likely to be the principal driver of the observed disparities, it is also feasible that treatment did not always occur in the location that was closest to where an individual lived—and that the occurrence of this phenomenon could feasibly differ between ethnicities. For example, the increased comorbidity burden among Māori may mean that Māori are more likely to be referred to high-volume centres for treatment; however, we might equally theorise that because Europeans with lung cancer likely face fewer economic barriers in terms of capacity for mobility (see NZDep findings, Supplementary Material 4), these patients may elect to travel to treatment centres outside of their region to undergo treatment. A separate, broader investigation of ethnic patterns in the likelihood of receiving cancer treatment in the location that is closest to home is needed to shed further light on this potential phenomenon; however, we remain confident that the primary driver of our observed differences in the current study is differences between Māori and Europeans in terms of where these population live.

## Conclusions

In a national study in a universal healthcare context, we found strong evidence of inequities in the travel required to access treatment for lung cancer. New Zealand’s Indigenous Māori population need to travel substantially further (for longer) to receive lung cancer surgery or radiation therapy, and these differences could not be explained by factors including age, sex, treatment type and comorbidity. Māori were also more likely to be treated within a high-volume hospital. Our findings bring into sharp focus the fact that centralisation of care may improve treatment outcomes, but it also makes accessing that treatment even more difficult for populations who are more likely to live rurally and in deprivation, such as Māori.

### Supplementary Information

Below is the link to the electronic supplementary material.

Supplementary file1 (DOCX 49 KB)

## Data Availability

Data cannot be shared publicly because of data restrictions put in place by the New Zealand Government. The data underlying the results presented in this study are available from the National Collections team at Te Whatu Ora – Health New Zealand, following a project review and approval process. For further information regarding access to these data, email data-enquiries@health.govt.nz.
