# Supplementary material for: Equity of travel to access surgery and radiation therapy for lung cancer in New Zealand
Source: Support Care Cancer. 2024 Feb 20;32(3):171. doi: 10.1007/s00520-024-08375-9 (PMC10879218; doi:10.1007/s00520-024-08375-9)
Supplement: Supplementary file 1 — Supplementary file1 (DOCX 49 KB) [file 520_2024_8375_MOESM1_ESM.docx]

**Supplementary Material 1:** Surgical procedure categories and individual procedures included in these categories.

| **Procedure Category** | **Name of Included Procedure** |
| --- | --- |
| **Curative**  Lobectomy  Pneumonectomy  Segmental Resection | Lobectomy of lung  Radical lobectomy  Pneumonectomy  Radical pneumonectomy  Endoscopic resection of bronchial tumour by laser  Segmental resection of lung  Wedge resection of lung  Endoscopic wedge resection of lung  Radical wedge resection of lung |
| Other Curative | Bronchoscopy with excision of lesion |
| **Palliative**  Drainage | Pericardiocentesis  Transthoracic drainage of pericardium  Subxyphoid drainage of pericardium  Thoracoscopic drainage of pericardium |
| Pleurodesis | Pleurodesis  Endoscopic pleurodesis |
| Other Palliative | Pulmonary decortication  Bronchoscopy with dilation  Pleurectomy  Insertion of tracheal stent |

**Supplementary Material 2:** Radiation therapy procedures, by data source.

| **Data Source** | **Name of Included Procedure** |
| --- | --- |
| **National Minimum Dataset**  NMDS; inpatient data | Brachytherapy with implantation  Intravascular brachytherapy  Stereotactic radiation therapy (SABR)  Hemi or total body irradiation |
| **National Non-Admitted Patient Collection** NNPAC; outpatient data | Orthovoltage attendance (PUC: M50024)  Megavoltage attendance (PUC: M50025)  Oncology-radiotherapy (PUC: M50005)  Oncology- stereotactic radiosurgery PUC: M50007)  Oncology- stereotactic radiotherapy (PUC: M50008) |

PUC – Purchase Unit Code, used to define procedures within the NNPAC.

**Supplementary Material 3:** Facilities, total procedure frequency and relevant hospital volume category, for lobectomy and segmental resection. Facility names have been withheld.

| **Facility Name** | *Total N (over 13 years)* | *n/year* | *%* | *Category* | *Rationale* |
| --- | --- | --- | --- | --- | --- |
| *Lobectomy* |  |  |  |  |  |
| Lobectomy Facility #1 | 1174 | 90 | 50% | High | ~1-2 per week |
| Lobectomy Facility #2 | 373 | 29 | 16% | Med | ~1-2 per month |
| Lobectomy Facility #3 | 295 | 23 | 13% | Med | ~1-2 per month |
| Lobectomy Facility #4 | 286 | 22 | 12% | Med | ~1-2 per month |
| Lobectomy Facility #5 | 183 | 14 | 8% | Med | ~1-2 per month |
| Lobectomy Facility #6 | 26 | 2 | 1% | Low | ~<=1-2 per year |
| Lobectomy Facility #7 | 3 | 0 | 0% | Low | ~<=1-2 per year |
| Lobectomy Facility #8 | 1 | 0 | 0% | Low | ~<=1-2 per year |
| Lobectomy Facility #9 | 1 | 0 | 0% | Low | ~<=1-2 per year |
| Lobectomy Facility #10 | 1 | 0 | 0% | Low | ~<=1-2 per year |
|  |  |  |  |  |  |
| *Segmental Resection* |  |  |  |  |  |
| Segmental Resection Facility #1 | 1155 | 89 | 36% | High | ~1-2 per week |
| Segmental Resection Facility #2 | 649 | 50 | 20% | Med | ~1-4 per month |
| Segmental Resection Facility #3 | 545 | 42 | 17% | Med | ~1-4 per month |
| Segmental Resection Facility #4 | 501 | 39 | 16% | Med | ~1-4 per month |
| Segmental Resection Facility #5 | 233 | 18 | 7% | Med | ~1-4 per month |
| Segmental Resection Facility #6 | 44 | 3 | 1% | Low | ~<=1-4 per year |
| Segmental Resection Facility #7 | 22 | 2 | 1% | Low | ~<=1-4 per year |
| Segmental Resection Facility #8 | 3 | 0 | 0% | Low | ~<=1-4 per year |
| Segmental Resection Facility #9 | 1 | 0 | 0% | Low | ~<=1-4 per year |
| Segmental Resection Facility #10 | 1 | 0 | 0% | Low | ~<=1-4 per year |
| Segmental Resection Facility #11 | 1 | 0 | 0% | Low | ~<=1-4 per year |
| Segmental Resection Facility #12 | 1 | 0 | 0% | Low | ~<=1-4 per year |

**Note:** ~ = approximately.

**Supplementary Material 4:** Characteristics of the cohort, by ethnicity.

|  | Total | **Māori** | | | **Pacific** | | | **Asian** | | | **MELAA/Other** | | | **European** | | |
| --- | --- | --- | --- | --- | --- | --- | --- | --- | --- | --- | --- | --- | --- | --- | --- | --- |
|  | *N* | *n* | *Crude %* | *Age Std. %* | *n* | *Crude %* | *Age Std. %* | *n* | *Crude %* | *Age Std. %* | *n* | *Crude %* | *Age Std. %* | *n* | *Crude %* | *Age Std. %* |
| **Total** | 27,869 | 5,601 | 20% | 24% | 1,267 | 5% | 5% | 1,180 | 4% | 5% | 123 | 0% | 0% | 19,698 | 71% | 66% |
| **Sex** |  |  |  |  |  |  |  |  |  |  |  |  |  |  |  |  |
| Female | 13,432 | 3,160 | 56% | 56% | 478 | 38% | 37% | 546 | 46% | 46% | 45 | 37% | 35% | 9,203 | 47% | 49% |
| Male | 14,437 | 2,441 | 44% | 44% | 789 | 62% | 63% | 634 | 54% | 54% | 78 | 63% | 65% | 10,495 | 53% | 51% |
| **Age** (years) |  |  |  |  |  |  |  |  |  |  |  |  |  |  |  |  |
| <50 | 1,089 | 337 | 6% |  | 102 | 8% |  | 122 | 10% |  | 12 | 10% |  | 516 | 3% |  |
| 50-64 | 7,115 | 2,310 | 41% |  | 416 | 33% |  | 350 | 30% |  | 40 | 33% |  | 3,999 | 20% |  |
| 65-74 | 9,206 | 1,913 | 34% |  | 406 | 32% |  | 371 | 31% |  | 30 | 24% |  | 6,486 | 33% |  |
| 75+ | 10,459 | 1,041 | 19% |  | 343 | 27% |  | 337 | 29% |  | 41 | 33% |  | 8,697 | 44% |  |
| **Deprivation** (NZDep decile) |  |  |  |  |  |  |  |  |  |  |  |  |  |  |  |  |
| 1-2 (least deprived) | 3,357 | 196 | 3% | 3% | 43 | 3% | 3% | 235 | 20% | 20% | 23 | 19% | 16% | 2,860 | 15% | 15% |
| 3-4 | 3,942 | 410 | 7% | 7% | 102 | 8% | 8% | 253 | 21% | 22% | 23 | 19% | 18% | 3,154 | 16% | 16% |
| 5-6 | 5,353 | 673 | 12% | 12% | 115 | 9% | 9% | 269 | 23% | 23% | 24 | 20% | 20% | 4,272 | 22% | 21% |
| 7-8 | 6,925 | 1,283 | 23% | 23% | 241 | 19% | 19% | 222 | 19% | 18% | 34 | 28% | 28% | 5,145 | 26% | 26% |
| 9-10 (most deprived) | 8,212 | 3,037 | 54% | 54% | 743 | 59% | 59% | 194 | 16% | 16% | 17 | 14% | 17% | 4,221 | 21% | 22% |
| Missing | 80 | 2 | 0% | 0% | 23 | 2% | 0% | 7 | 1% | 0% | 2 | 2% | 0% | 46 | 0% | 0% |
| **Rurality** (GCH Category) |  |  |  |  |  |  |  |  |  |  |  |  |  |  |  |  |
| Urban 1 | 14,902 | 2,387 | 43% | 43% | 1,104 | 87% | 87% | 1,081 | 92% | 91% | 100 | 81% | 84% | 10,230 | 52% | 52% |
| Urban 2 | 6,129 | 1,478 | 26% | 26% | 66 | 5% | 5% | 45 | 4% | 4% | 10 | 8% | 5% | 4,530 | 23% | 23% |
| Rural 1 | 4,175 | 868 | 15% | 15% | 46 | 4% | 3% | 37 | 3% | 3% | 7 | 6% | 6% | 3,217 | 16% | 16% |
| Rural 2 | 2,065 | 653 | 12% | 12% | 17 | 1% | 2% | 5 | 0% | 0% | 3 | 2% | 3% | 1,387 | 7% | 7% |
| Rural 3 | 403 | 198 | 4% | 4% | 3 | 0% | 0% | 2 | 0% | 0% | - | 0% | 0% | 200 | 1% | 1% |
| Missing | 195 | 17 | 0% | 0% | 31 | 2% | 0% | 10 | 1% | 0% | 3 | 2% | 0% | 134 | 1% | 0% |
| **Comorbidity** (C3 Index score) | |  |  |  |  |  |  |  |  |  |  |  |  |  |  |  |
| <=0 | 13,382 | 2,593 | 46% | 46% | 665 | 52% | 54% | 783 | 66% | 68% | 73 | 59% | 59% | 9,268 | 47% | 54% |
| 0-1 | 3,672 | 644 | 11% | 11% | 116 | 9% | 9% | 130 | 11% | 11% | 16 | 13% | 13% | 2,766 | 14% | 14% |
| 1-2 | 4,116 | 908 | 16% | 16% | 169 | 13% | 13% | 111 | 9% | 9% | 12 | 10% | 10% | 2,916 | 15% | 14% |
| >2 | 6,699 | 1,456 | 26% | 26% | 317 | 25% | 24% | 156 | 13% | 11% | 22 | 18% | 18% | 4,748 | 24% | 18% |
| **Tumour Type** |  |  |  |  |  |  |  |  |  |  |  |  |  |  |  |  |
| Small Cell | 3,025 | 906 | 16% | 16% | 116 | 9% | 10% | 66 | 6% | 6% | 6 | 5% | 5% | 1,931 | 10% | 11% |
| Non-Small Cell | 18,107 | 3,549 | 63% | 63% | 921 | 73% | 75% | 960 | 81% | 83% | 96 | 78% | 78% | 12,581 | 64% | 69% |
| Other/Unspecified | 6,737 | 1,146 | 20% | 20% | 230 | 18% | 16% | 154 | 13% | 11% | 21 | 17% | 17% | 5,186 | 26% | 20% |
| **Stage** |  |  |  |  |  |  |  |  |  |  |  |  |  |  |  |  |
| Local | 1,779 | 244 | 4% | 4% | 68 | 5% | 6% | 131 | 11% | 12% | 8 | 7% | 6% | 1,328 | 7% | 8% |
| Regional | 3,681 | 821 | 15% | 15% | 161 | 13% | 13% | 204 | 17% | 18% | 23 | 19% | 22% | 2,472 | 13% | 14% |
| Advanced | 12,614 | 2,515 | 45% | 45% | 703 | 55% | 56% | 557 | 47% | 48% | 54 | 44% | 44% | 8,785 | 45% | 47% |
| Unstaged | 9,795 | 2,021 | 36% | 36% | 335 | 26% | 25% | 288 | 24% | 22% | 38 | 31% | 29% | 7,113 | 36% | 30% |

Age Std. – age standardised.

**Supplementary Material 5:** Receipt of surgery and radiation therapy, for all ethnic groups.

|  | **Māori** | | | **Pacific** | | | **Asian** | | | **MELAA/Other** | | | **European** | | |
| --- | --- | --- | --- | --- | --- | --- | --- | --- | --- | --- | --- | --- | --- | --- | --- |
| **Treatment** | *n* | *%* | *Age Std. %* | *n* | *%* | *Age Std. %* | *n* | *%* | *Age Std. %* | *n* | *%* | *Age Std. %* | *n* | *%* | *Age Std. %* |
| Received Any Primary Surgery | 745 | 13% | 13% | 209 | 16% | 17% | 307 | 26% | 27% | 23 | 19% | 19% | 3073 | 16% | 19% |
|  |  |  |  |  |  |  |  |  |  |  |  |  |  |  |  |
| Received Any Curative Surgery | 522 | 9% | 9% | 118 | 9% | 10% | 219 | 19% | 19% | 20 | 16% | 18% | 2264 | 11% | 14% |
| Lobectomy | 345 | 6% | 6% | 96 | 8% | 8% | 184 | 16% | 16% | 12 | 10% | 9% | 1510 | 8% | 9% |
| Segmental Resection | 163 | 3% | 3% | 25 | 2% | 2% | 40 | 3% | 3% | 5 | 4% | 5% | 725 | 4% | 4% |
| Pneumonectomy | 37 | 1% | 1% | 6 | 0% | 0% | 11 | 1% | 1% | 3 | 2% | 3% | 161 | 1% | 1% |
| Other Curative | 4 | 0% | 0% | 2 | 0% | 0% | 3 | 0% | 0% | 0 | 0% | 0% | 18 | 0% | 0% |
|  |  |  |  |  |  |  |  |  |  |  |  |  |  |  |  |
| Received Any Palliative Surgery | 241 | 4% | 4% | 93 | 7% | 7% | 91 | 8% | 8% | 3 | 2% | 1% | 881 | 4% | 5% |
| Drainage | 76 | 1% | 1% | 18 | 1% | 1% | 26 | 2% | 2% | 0 | 0% | 0% | 177 | 1% | 1% |
| Pleurodesis | 157 | 3% | 3% | 73 | 6% | 6% | 70 | 6% | 6% | 3 | 2% | 1% | 685 | 3% | 4% |
| Other Palliative | 18 | 0% | 0% | 4 | 0% | 0% | 1 | 0% | 0% | 0 | 0% | 0% | 49 | 0% | 0% |
|  |  |  |  |  |  |  |  |  |  |  |  |  |  |  |  |
| Received Any Radiation Therapy | 2446 | 44% | 44% | 461 | 36% | 38% | 381 | 32% | 33% | 52 | 42% | 42% | 7620 | 39% | 44% |
|  |  |  |  |  |  |  |  |  |  |  |  |  |  |  |  |
